# Reconsidering Semaglutide Use for Chronic Obesity in Patients of Asian Descent: A Critical Review

**DOI:** 10.7759/cureus.73111

**Published:** 2024-11-06

**Authors:** Jenny Lu, Grace Williams, Stacey Fanning

**Affiliations:** 1 Medicine, Touro College of Osteopathic Medicine, New York, USA; 2 Immunology, Touro College of Osteopathic Medicine, New York, USA

**Keywords:** asian american, bmi, diabetes, disparities, health public, metabolic syndrome, minority health, minority population, obesity, visceral fat

## Abstract

Semaglutide is a glucagon-like peptide 1 (GLP-1) receptor agonist. It is the first approved drug for chronic weight management in adults who are overweight or obese since 2014. Its increasing popularity has garnered significant media attention and led to a drug shortage, resulting in limited access for its intended use - patients with type 2 diabetes. Numerous studies have demonstrated its effectiveness in promoting weight loss. This review seeks to explain the use of semaglutide, a GLP-1 receptor agonist, to treat metabolic syndrome in the Asian American population. It raises concerns about the existing diagnostic and treatment approaches and stresses the necessity of integrating visceral fat and other ethnicity-specific risk predictors for the diagnosis of metabolic syndrome. The objective of this review is to examine the eligibility criteria for the prescription of semaglutide critically and determine whether Asians are being unfairly excluded and denied access to this medication due to ineffective prescription guidelines.

## Introduction and background

Metabolic syndrome is a global concern, affecting one-fourth of the adult population worldwide [1]. Metabolic syndrome, also known as insulin resistance syndrome, is characterized by abdominal obesity (waist size >35 inches for women, >40 for men) and two of the three following criteria: high blood pressure, impaired glucose metabolism, elevated non-high-density lipoprotein (non-HDL), and cholesterol level (atherogenic dyslipidemia) [2,3]. However, some data suggest that these criteria may not effectively identify metabolic risk, particularly in some ethnic groups [3]. Of these populations, Asian Americans are perhaps the most difficult to identify and manage for metabolic syndrome [4]. Asian Americans have been shown to have normal or lower BMI than other ethnic groups, but show a high incidence of type 2 diabetes and other symptoms of metabolic syndrome when compared to other race groups [5-8]. 

One possible explanation for this trend is the disparity in the body fat or adiposity distribution of persons of Asian descent [9]. Researchers have noted that Asians have higher amounts of visceral fat at lower BMIs than their European descent counterparts [5,6]. It has been shown that abdominal obesity or, more specifically, the amount of visceral fat is more closely linked to insulin resistance, an abnormal lipid profile, and other features of metabolic syndrome. Therefore, other criteria in addition to BMI should be used for estimating metabolic risk.

Semaglutide is a GLP-1 receptor agonist currently approved for type 2 diabetes and chronic obesity in adults. Since 2014, the criteria for prescribing semaglutide have been based on BMI. However, if Asian Americans exhibit different BMI values despite having higher levels of visceral body fat and instances of metabolic syndrome at lower BMIs, it may be necessary to reconsider BMI as the primary indication for this medication.

Purpose of the study

This critical review aims to assess the effectiveness of semaglutide and its applicability to the management of metabolic syndrome among Asian Americans. Through a review of the literature and analysis of clinical data, it aims to pinpoint shortcomings in current diagnostic and treatment models and suggests more suitable approaches to meet the metabolic health needs of Asian Americans. The discussion revolves around the possible advantages of semaglutide treatment, the drawbacks of using BMI parameters, and the necessity of considering ethnic factors when diagnosing and treating metabolic syndrome.

## Review

Methodology

This research incorporated a critical literature review that aimed at providing a critical assessment of the possibility of using semaglutide for managing metabolic syndrome for Asian Americans. The electronic databases employed for this work comprised of PubMed, as well as academic journals and articles. Specific terms searched were: "metabolic syndrome", "Asian Americans", "body mass index", "visceral fat", "semaglutide", and other relevant words. Some of the advanced search options involved the use of Boolean operators such as ‘AND’ and ‘OR’ to search for a specific term together with the related terms. Studies published within two decades from 2004 to 2024 were included. Only studies published in English were considered. The search was refined to include only clinical studies and trials, comparative studies, multicenter studies, observational studies, and randomized controlled trials. Only free full-text articles were included. Preprints were excluded to ensure the inclusion of peer-reviewed studies only. All the peer-reviewed studies containing meaningful data are critically reviewed.

Indicators of metabolic syndrome in patients 

BMI and Metabolic Syndrome in Asian Americans

Asian American patients frequently exhibit metabolic syndrome or its components, such as abdominal obesity, hypertension, impaired fasting glucose, elevated triglycerides, or low HDL cholesterol, despite having a normal BMI. This phenomenon, often termed "normal-weight obesity" or "thin outside, fat inside" (TOFI), reflects an increased proportion of visceral adiposity, which is a key contributor to metabolic risk [3]. Several studies done in the literature have shown that Asian Americans have a significantly higher prevalence of diabetes and metabolic syndrome even though their BMI is found to be slightly lower than other ethnicities [10,11]. This gave rise to what has been referred to as the "Asian paradox" [12,13], which helps support the argument that BMI is not a reliable marker of metabolic health in Asians. Thus, increased body fat residing in specific locations, even at normal BMIs, could contribute substantially to metabolic changes in Asian Americans, including a greater proportion of visceral fat at lower weights. 

There are many critiques of using BMI to measure health. For example, BMI has long been criticized for not capturing appropriate short-term changes during the growth phases in children and adolescents or during phases of muscle wasting among the elderly [14]. BMI does not reflect visceral adiposity, which is paramount in metabolic disorders and cardiovascular conditions. In addition, gender differences play a significant role, as men typically have a lower percentage of body fat and higher lean mass compared to women [15]. These factors collectively highlight that BMI is not an ideal tool for accurately measuring adiposity and metabolic health. This further suggests that, in addition to being Asian, BMI is not a suitable criterion. Being an Asian woman, in particular, may be adversely affected by the use of BMI as the sole qualifying factor for the approval of semaglutide for weight loss.

Visceral Fat and Metabolic Syndrome

Visceral fat, particularly in the abdominal region, plays a significant role in influencing insulin resistance, dyslipidemia, and other features of metabolic syndrome [16]. Several studies have shown that visceral fat is causatively linked to metabolic syndrome through various mechanisms. Excess visceral fat becomes dysfunctional and releases adipokines and inflammatory cytokines, such as tumor necrosis factor-alpha (TNF-α) and interleukin-6 (IL-6), which cause chronic low-grade inflammation and insulin resistance-key characteristics of type 2 diabetes [17,18].

Enlarged adipose tissue from accumulated visceral fat releases free fatty acids (FFAs) into the portal circulation, leading to hepatic insulin resistance, elevated blood sugar levels, and dyslipidemia, which manifests as high triglycerides and low high-density lipoprotein (HDL) cholesterol levels. Excess FFAs, reactive oxygen species (ROS), and pro-inflammatory cytokines are released from these tissues. When these excess FFAs and dietary lipids accumulate in non-adipose organs such as the liver, muscle, and pancreas, they are deposited as ectopic fat, causing lipotoxicity. This lipotoxicity disrupts the function of cellular organelles like mitochondria, the endoplasmic reticulum, and lysosomes, releasing excess ROS and pro-inflammatory factors, which in turn cause systemic inflammation [19,20].

Chronic low-grade systemic inflammation impairs insulin's action in the signaling pathway, disrupting glucose homeostasis and resulting in systemic dysregulation. Over time, visceral fat-induced insulin resistance forces the pancreas to secrete more insulin to maintain normal blood glucose levels [21]. However, this compensatory hyperinsulinemia eventually becomes insufficient, leading to elevated blood glucose levels and the development of type 2 diabetes. Insulin resistance also impairs the suppression of lipolysis in adipose tissue, increasing FFAs in the blood and further exacerbating metabolic disturbances [16,22,23].

Semaglutide therapy and metabolic syndrome

Overview of Semaglutide

Semaglutide is a glucagon-like peptide-1 (GLP-1) receptor agonist used to manage type 2 diabetes and obesity [24]. It is a synthetic analog of the naturally occurring hormone GLP-1, which is essential for glucose metabolism and appetite regulation [24,25]. By mimicking GLP-1, semaglutide enhances glycemic control and promotes weight loss through several mechanisms that target metabolic processes involved in metabolic syndrome.

Semaglutide works primarily by enhancing glucose-stimulated insulin secretion from pancreatic beta cells [26] and increasing glucose-dependent insulin release, thereby improving blood glucose regulation [27]. In addition, it inhibits glucagon secretion from pancreatic alpha cells. Glucagon is a hormone that typically raises blood glucose levels by stimulating glycogenolysis and gluconeogenesis in the liver. By reducing glucagon secretion, semaglutide decreases gluconeogenesis, helping to lower blood glucose levels further [28-29].

Another key effect of semaglutide is its ability to slow gastric emptying, which reduces the rate at which nutrients enter the bloodstream and prevents sharp increases in postprandial glucose levels [30]. This helps maintain more stable blood glucose levels after meals. In addition, semaglutide reduces food intake by enhancing satiety, thereby supporting weight loss, especially in individuals with metabolic syndrome [31].

Efficacy of Semaglutide in Metabolic Syndrome

Clinical studies have demonstrated the efficacy of semaglutide in managing various components of metabolic syndrome, including hyperglycemia, obesity, dyslipidemia, and hypertension [32]. 

Semaglutide has been shown to significantly reduce patient's HbA1c levels with type 2 diabetes [22]. Randomized controlled clinical studies showed that patients on semaglutide had significant changes in HbA1c compared to placebo or other antidiabetic drugs [33-35]. Semaglutide has shown promising and substantiated weight loss outcomes for both diabetic and non-diabetic patients [25,36-37]. Semaglutide use has also been shown to decrease triglyceride levels and increase the level of high-density lipoprotein cholesterol [38,39]. While GLP-1 receptor agonists (GLP-1RAs) improve glycemic control and aid in weight loss, semaglutide’s safety profile has been extensively evaluated through phase 3 trials, including cardiovascular outcome studies for both subcutaneous (SUSTAIN) and oral (PIONEER) forms. Despite approval for long-term use, the duration of risk remains uncertain, warranting continued research. Reported adverse events include hypoglycemia, gastrointestinal issues, pancreatic and thyroid cancer risks, gallbladder and cardiovascular events, acute kidney injury, diabetic retinopathy complications, and injection-site or allergic reactions. Increased triglyceride and high levels of HDL changes are positive for cardiovascular risk, a critical component in metabolic syndrome management [33-39]. The weight reduction promoted by semaglutide is seen to contribute to the decrease of systolic and diastolic blood pressure most likely due to decreased body mass [40,41]. In addition to changes in metabolic variables, there is evidence supporting the efficacy of semaglutide in reducing cardiovascular events [42-44]. 

Semaglutide Use for Metabolic Syndrome in Patients of Asian Descent

There may be pharmacogenetic disparities in drug responses and adverse reactions to drugs among the Asian populations [45]. Such differences stem possibly from the genetic variations of pharmacokinetics and pharmacodynamics of drug metabolism, effectiveness, and toxicity, owing to genetic changes in genes such as CYPs and drug transporters [46]. Although there seem to be differences in the drug metabolism of some drugs, at this time, there remain inconclusive data regarding the efficacy of using a different dosage of semaglutide for specific Asian subgroups.

Recent clinical trials have been documented in Asians and its efficacy in weight reduction and controlling symptoms of metabolic syndrome. The STEP (Semaglutide Treatment Effect in People with Obesity) clinical trial, which included an East Asian population with and without diabetes, provided data that semaglutide use led to a significant increase in weight loss compared to the placebo group. The mean weight loss from baseline to week 68 was 13.2% with semaglutide 2.4 mg and 9.6% with semaglutide 1.7 mg. A larger proportion of participants achieved ≥5%, ≥10%, and ≥15% reduction in baseline body weight with semaglutide 2.4 mg compared to placebo (83% vs. 21%, 61% vs. 5%, and 41% vs. 3%, respectively) [47,48]. The SUSTAIN study (Semaglutide Unabated Sustainability in Treatment of Type 2 Diabetes) provides data showing that Asian patients with DM2 can benefit from semaglutide [49]. Chinese participants who were treated with semaglutide had higher odds of reaching target HbA1c levels below 7.0% (53 mmol/mol), achieving weight losses of 5% or more and 10% or more, and meeting combined goals of improved HbA1c without severe hypoglycemia or weight gain. The PIONEER 10 study indicated that oral semaglutide was well tolerated by Japanese patients with type 2 diabetes, significantly reducing HbA1c (14 mg dose) and body weight (7 and 14 mg doses) [50,51]. Significant decreases in weight, BMI, fasting blood glucose, HbA1c, and medication effect score were observed from baseline to follow-up (-4.0 kg, -1.5 kg/m², -23.1 mg/dl, -1.2%, -0.4, respectively). 

These trials show that semaglutide is an effective modulator of metabolic syndrome, providing strong evidence of its efficacy amongst Asians in the treatment of diabetes and chronic weight management (Table 1).

**Table 1:** Summary table of studies included in the present systemic literature review and narrative description.

Study details	Study design	Target group	Outcome measure	Summary of findings	Citations
Zhu et al. (2021)	Cross-sectional study	Data was pulled from NHANES (National Health and Nutrition Examination Survey) 2011-2016 data, which included: a home interview, a complete health examination survey, and then a standardized health examination at a mobile examination center. Subjects were Asian Americans (n=2121), non-Hispanic White people (n=6318) (total n=8439).	The prevalence of metabolic syndrome and its individual components for three BMI categories were examined. Multivariate binary logistic regression was used to compare the risk of metabolic syndrome in Asian Americans to non-Hispanic White people.	Asian Americans, both men and women, had a higher prevalence of metabolic syndrome with BMI > 23 than non-Hispanic White people of the same BMI level.	[4]
Palaniappan et al. (2010)	Cross-sectional study	Electronic health records of primary care patients, aged 35 or older, with self-identified race/ethnicity (Asian Indian, Chinese, Filipino, Japanese, Korean, Vietnamese, or non-Hispanic White) in San Francisco Bay Area from January 1, 2006-December 31, 2008 (n=43507).	Patient demographics and characteristics were described using means and proportions. Means were compared using ANOVA (analysis of variance) and turn-key comparisons. Metabolic syndrome prevalence rates across different Asian subgroups and BMI categories were compared.	Although Asian Americans had lower rates of BMI values, they had higher rates of metabolic syndrome compared to non-Hispanic White people.	[5]
Katz et al. (2013)	Longitudinal study	Data was pulled from three prospective, observational studies: PRC (People's Republic of China) Study, CARDIA (Coronary Artery Risk Development in Young Adults) Study, and ARIC (Atherosclerosis Risk in Communities) Study The study populations included young (24-39 years) and middle-aged (45-64 years) subjects whose ethnic make-up included: Chinese Americans (n=5354), American Blacks (n=6076), and American White people (n=13451) (total n=24881).	BMI and incident hypertension were examined over approx. 9.5 years. Risk factors were estimated using logistic regression models and calculated adjusted incidences and incidence differences. Ethnic-specific differences in BMI were also examined.	The association of BMI with incident hypertension was steeper in Chinese (p<0.05) than in American populations during young and middle adulthood.	[7]
McNeely et al. (2004)	National population-based telephone survey	Telephone survey of health status and health behaviors of Americans in all 50 states, Guam, Puerto Rico, and U.S. Virgin Islands. Subjects included: 3071 Asians, 12561 blacks, 12153 Hispanics, 2299 Native Americans, 626 Pacific Islanders, and 129116 non-Hispanic White people, all >30 years (n=159826).	Subjects were assumed to have physician-diagnosis of type 2 diabetes unless diagnosed before age 30; age, BMI, and sex were adjusted for.	Similar proportions of Asian and non-Hispanic white Americans reported a diabetes diagnosis. However, after accounting for a lower BMI in Asians, the adjusted prevalence of diabetes is 60% higher in Asian Americans.	[8]
Nakagami et al. (2003)	Cross-sectional study	Data from population-based studies conducted after 1980 from the DECODE (The Diabetes Epidemiology: Collaborative Analysis of Diagnostic Criteria in Europe) and DECODA (The Diabetes Epidemiology: Collaborative Analysis of Diagnostic Criteria in Asia) studies that represented different ethnic groups: 11 European, 1 Maltese, 3 Indian, 2 Chinese, and 3 Japanese surveys. That included 14,240 men and 15,129 women from ages 30 to 89 (n=29369).	The prevalence of diabetes by age and sex was stratified by ethnic group as was BMI.	The prevalence of diabetes was higher in the Indian and Maltese studies compared to Japan, China, and Europe. BMI had lower thresholds in Indian and Maltese subjects with age-adjusted Type 2 diabetes compared to those subjects in Europe. This difference should be reflected in "optimal" BMI recommendations for these populations.	[11]
Santorelli et al. (2021)	Cross-sectional study	225 white and 269 South Asian children from the Born in Bradford cohort study (n= 541).	Anthropometric measurements and DXA (dual-energy X-ray absorptiometry) scans were taken from 541 white and South Asian children. Linear regression was used to examine total body fat percent and total and regional fat mass by ethnicity.	There may be a relationship between greater total body fat percentage and regional fat mass in South Asian children compared to white children. This suggests possible future cardiometabolic disease at a BMI level that is below the obesity threshold.	[13]
Lee et al. (2020)	Longitudinal cohort study	Korean adults, aged 18 to 65, who were metabolically healthy and had a normal weight, and had received health screenings over a 3-year follow-up period, at Seoul National University Hospital (n=3093).	Cox proportional hazard models were used to estimate adjusted hazard ratios and 95% confidence intervals for incident metabolic syndrome, increase in visceral adipose tissue, and body mass index.	A longitudinal relationship was found between visceral obesity and obesity and an increased risk of metabolic syndrome in metabolically healthy adults. Visceral fat also appeared to be a better predictor of metabolic syndrome.	[16]
Kadowaki et al. (2022)	Interventional: randomized, double-blind, double-dummy, placebo-controlled, phase 3a superiority trial	Adults from East Asia, specifically > 18 years in South Korea or >20 years in Japan, with a BMI of at least 27·0 kg/m2 with two or more weight-related comorbidities or a BMI of 35·0 kg/m2 or more with one or more weight-related comorbidity, who had at least one self-reported unsuccessful dietary attempt to lose body weight (n= 437 screened, n=401 enrolled).	Percentage change in body weight from baseline to week 68 and the proportion of participants who had achieved a reduction of at least 5% of baseline body weight at week 68.	A larger proportion of East Asian adults with obesity, with or without type 2 diabetes, who were given semaglutide 2·4 mg once a week, had a 5% or higher reduction in body weight compared to the placebo group. This study had clinically meaningful reductions in body weight and abdominal visceral fat compared to placebo.	[47]
Mu et al. (2024)	Interventional: randomised phase 3a, double-blind multicentre controlled trial	Adults from East Asia specifically from China, Hong Kong, Brazil, and South Korea, who were overweight or obese, with or without type 2 diabetes (n= 448 screened, n=375 enrolled).	Mean percentage change in body weight from baseline to week 44.	At week 44, the proportion of participants who used semaglutide 2·4 mg once a week and lost 5% or more of their body weight, was higher in the semaglutide 2·4 mg group compared to the placebo. These results supported semiglutide use in East Asian ethnic groups who were overweight or obese and with or without type 2 diabetes.	[48]
Ji et al. (2020)	Randomized, double-blind, double-dummy, active-controlled, multicentre and multiregional, parallel-group trial	East Asian patients in China, the Republic of Korea, Brazil, South Africa, and Ukraine, >18 years or older, diagnosed with type 2 diabetes with HbA1c 7.0%-10.5% (53-91 mmol/mol), and treated with metformin monotherapy at a stable dose of 1500 mg or higher (n=868 screened, n=605 enrolled).	Percentage change in body weight from baseline to week 30; the proportion of participants who at week 30 achieved targets of HbA1c less than 7.0% (53 mmol/mol); participants who achieved higher than weight loss of 5%.	Semaglutide was superior to sitagliptin in improving glycemic control and reducing body weight in patients with type 2 diabetes that was inadequately controlled on metformin.	[49]
Yabe et al. (2022)	Phase 2/3a, randomized, placebo- and active-controlled trial	Japanese adults aged >20 years, diagnosed with type 2 diabetes at least 30 days before screening, had an HbA1c of 6.5-9.5% if also receiving background oral glucose-lowering medication as monotherapy or 7.0-10.0% if treated with medical nutritional therapy and exercise alone (n=701 enrolled).	Change in HbA1c from baseline to week 26, change in body weight from baseline to week 26.	Semaglutide had dose-dependent HbA1c decreases in Japanese patients with type 2 diabetes.	[50]
Wajid et al. (2023)	Ambi-directional cohort study	Pakistani patients, aged 18 -65, who were diagnosed with type 2 diabetes and were prescribed semaglutide and then took it for at least 3 months (n=135 enrolled).	A chart review was made for all patients with type 2 diabetes who were prescribed semaglutide with baseline data recorded. After three months, the following was recorded: HbA1c, BMI, adverse effects profile, treatment satisfaction questionnaire for medications (TSQM-9) was administered, and medication effect score.	The mean differences in weight, BMI, fasting blood glucose, HbA1c, and medication effect score decreased after three months after semaglutide use. Semaglutide was shown to be effective in decreasing HbA1c and body weight.	[52]

Limitations of the Current Diagnostic Criteria

Currently, the only semaglutide injection that is FDA-approved for weight loss in adults is Wegovy. The criteria for weight loss use is listed for adults with a BMI ≥ 30 or BMI ≥ 27 with additional weight-related medical problems. This may inadvertently exclude Asian Americans who are at significant metabolic risk but do not meet the conventional BMI thresholds [52]. This raises concerns regarding equity in access to effective treatments for metabolic syndrome within this population. The reliance on traditional BMI thresholds without including other factors such as waist circumference measurements in diagnosing metabolic syndrome may result in underdiagnosis and undertreatment of at-risk individuals. Ethnic-specific differences in body composition and fat distribution underscore the need for a more nuanced approach to defining metabolic risk [53]. Alternative measures, such as incorporating visceral fat assessment and modifying BMI thresholds specific to ethnicity, may enhance the accuracy of identifying individuals at metabolic risk in diverse populations.

Overall, the literature underscores the complexity of metabolic health disparities in Asian Americans and highlights the inadequacy of current diagnostic and treatment paradigms. Addressing these disparities requires a multifaceted approach that considers ethnic-specific differences in body composition, incorporates alternative measures of adiposity and fat distribution, and ensures equitable access to effective treatments such as semaglutide.

Future directions for research

While the literature review provides valuable insights into the relationship between metabolic syndrome, BMI, visceral fat, and semaglutide therapy in Asian Americans, several research directions for further investigations are specified below. There is a need for additional studies that seek to identify new predictors of eligibility for the therapy by semaglutide and the effects of incorporating measurements of visceral obesity on its efficacy in patients of Asian descent with metabolic syndrome. Thus, the discussion indicates the necessity to address the problem of diagnosing and treating metabolic syndrome in Asian Americans with more sensitivity. Healthcare professionals and policymakers should pay attention to ethnic variations in anthropometry, body composition, and fat topography to ensure the optimum delivery of quality intervention for metabolic syndrome in this ethnic group.

## Conclusions

The literature review has revealed that it is insufficient to use BMI as the primary criterion for approval of semaglutide for chronic obesity and metabolic syndrome. This leads to excluding Asian Americans from being properly treated with GLP-1 analogs for obesity. Visceral fat is key in evaluating metabolic health as that is one of the defining criteria of metabolic syndrome. In addition, we have found evidence that Asians amass more central adiposity at lesser BMI levels than other races. This study implicates the necessity of a broader understanding of diagnosing and treating metabolic syndrome in the Asian-American population. By implementing accurate diagnostic and treatment methods, clinicians and policymakers can enhance the inclusion of Asian American patients who could benefit from the drug but are currently excluded due to criteria such as BMI, which inadequately measures metabolic syndrome.
